# Enhanced Sorption Performance of Natural Zeolites Modified with pH-Fractionated Humic Acids for the Removal of Methylene Blue from Water

**DOI:** 10.3390/molecules28207083

**Published:** 2023-10-14

**Authors:** Stefano Salvestrini, Jean Debord, Jean-Claude Bollinger

**Affiliations:** 1 Department of Environmental, Biological and Pharmaceutical Sciences and Technologies, University of Campania “Luigi Vanvitelli”, 81100 Caserta, Italy; 2 Service de Pharmacologie-Toxicologie, Hôpital Dupuytren, 87042 Limoges, France; jean.debord@unilim.fr; 3 Laboratoire E2Lim, Faculté des Sciences et Techniques, Université de Limoges, 87060 Limoges, France; jean-claude.bollinger@unilim.fr

**Keywords:** adsorption, sorption, humic acids, pH fractionation, natural zeolites, methylene blue

## Abstract

This work explores the effect of humic acids (HA) fractionation on the sorption ability of a natural zeolite (NYT)—HA adduct. HA were extracted from compost, fractionated via the pH fractionation method, and characterized via UV-Vis spectroscopy and gel permeation chromatography. The HA samples were immobilized onto NYT via thermal treatment. The resulting adducts (NYT-HA) were tested for their ability to remove methylene blue (MB) from an aqueous solution. It was found that the sorption performance of NYT-HA strongly depends on the chemical characteristics of humic acids. Sorption capacity increased with the molecular weight and hydrophobicity degree of the HA fractions. Hydrophobic and π–π interactions are likely the primary mechanisms by which MB interacts with HA. The sorption kinetic data conform to the pseudo-second-order model. The Freundlich isotherm model adequately described the sorption equilibrium and revealed that the uptake of MB onto NYT-HA is endothermic in nature.

## 1. Introduction

Over the years, the continued release of organic contaminants from anthropogenic activities into natural waters has attracted increased attention owing to its implications for human health and environmental preservation [1].

Various techniques for the abatement of pollutants in water have been investigated. Among them, innovative processes using reactive oxygen species as degrading agents of pollutants (Advanced Oxidation Processes, AOPs) are currently being investigated [2,3,4,5]. Although AOPs are very promising, the well-established process of sorption is still extensively studied in the field of water remediation, mostly because of its effectiveness and ease of use [6].

Different materials have been tested as sorbents in aqueous systems. Activated carbon is probably the most widely applied sorbent for organic compounds because of its high surface area and low water affinity [7]. In the last few decades, the development of alternative sorbents, especially naturally occurring materials, has intensified. Of particular interest are natural minerals coated with an appropriate organic phase that imparts enhanced sorption ability to original materials. Among these minerals, organo-modified natural zeolites are noteworthy due to their pollutant removal efficiency. The organo-modification of zeolites can be accomplished using the following approaches: (i) the impregnation method, in which a zeolite is treated with a solution of a desired compound; (ii) the replacement of external exchangeable elemental cations (e.g., Na^+^, K^+^, and Ca^2+^) with organic cations; and (iii) the direct interaction of the external exchangeable cations with negatively charged molecules. Examples of the first approach include the use of biochar [8], chitosan [9], and biological matter [10,11]. The cation exchange modification of zeolites can be achieved using quaternary ammonium salts such as hexadecyltrimethylammonium chloride [12], cetylpyridinium bromide [13], and stearyldimethylbenzylammonium chloride [14,15]. An example of the third type of modification involves the use of humic acids (HA) [16,17]. Thanks to their chemical heterogeneity (e.g., large mass distribution, presence of various functional groups, and presence of hydrophilic and hydrophobic moieties [18,19,20,21]), HA are able to efficiently interact with a wide variety of chemical compounds, rendering the zeolite-HA adduct a very versatile sorbent [22]. The loading of HA on zeolites is favored in the presence of exchangeable Ca^2+^ ions, which not only act as a bridge between the negatively charged surface of zeolites and the deprotonated functional groups (mainly carboxylic) of HA [17] but also promote HA self-aggregation [23].

Interestingly, the sorptive performance of the zeolite-HA material can be improved by selectively employing only the more appropriate fraction of a raw HA sample. HA fractionation can be achieved by means of ultrafiltration [24] or pH adjustments [25]. In particular, pH fractionation is preferred over ultrafiltration because it is easier to execute and does not suffer from the progressive fouling of the filter. The various pH-fractionated HA samples are expected to have different chemical compositions [25,26] and possibly different affinities for the sorbable organic contaminants.

Based on the above considerations, in this study, an HA sample was extracted from a commercial vegetable compost and fractionated using the pH fractionation method. The unfractionated HA and their fractions were characterized via UV-Vis spectroscopy and gel permeation chromatography. The samples were immobilized on a natural zeolite (Neapolitan Yellow Tuff, NYT) and tested with respect to their ability to act as sorbents for the removal of methylene blue (MB). MB was chosen as a model compound due to its high occurrence in water worldwide and its harmfulness to human health and the environment [27,28]. The kinetic and thermodynamic aspects of the sorption process were also investigated.

## 2. Results and Discussion

### 2.1. HA Characterization

**Figure 1:**
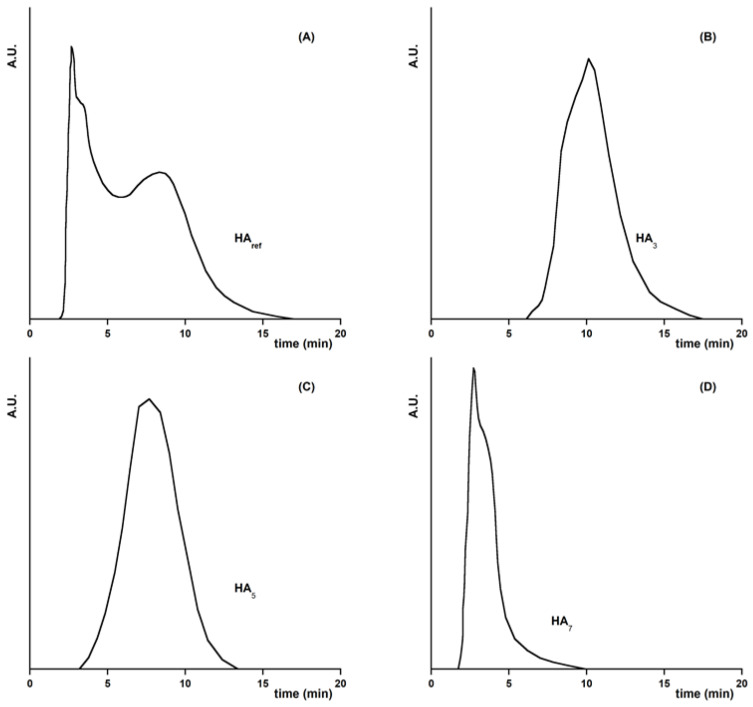
High-pressure gel permeation chromatograms of extracted HA samples: (**A**) unfractionated HA; (**B**) fraction of HA soluble at pH 3; (**C**) fraction of HA insoluble at pH 3 and dissolved at pH 5; (**D**) residual fraction of HA insoluble at pH 5 and dissolved at pH 7.

The HA used for this study were extracted from vegetable compost and fractionated via sequential pH solubilization. To gain information on the physico-chemical characteristics of the HA, high-pressure gel permeation chromatography analyses were performed [29]. The results of these experiments are reported in Figure 1. The chromatogram of the unfractionated HA (HA_ref_, Figure 1A) is very broad, typical of polydisperse substances with a wide distribution of molecular weights [30]. In contrast, all the pH-fractionated HA samples (Figure 1B–D) exhibit a slightly more symmetrical and sharp elution peak, indicating the presence of a more homogeneous distribution of molecules with respect to HA_ref_. Moreover, the retention time of the pH-fractionated HA decreases with an increasing pH. This suggests that the HA samples dissolved at higher pH levels have a higher average molecular weight. The latter finding is in agreement with the results obtained by Zhang et al. using pH-fractionated HA derived from Chinese weathered coal [25]. Additional information on the characteristics of the HA fractions were obtained via UV-Vis spectroscopic measurements, specifically by determining the ratio of absorbance at 250 nm to 365 nm (E_2_/E_3_) and at 465 nm to 665 nm (E_4_/E_6_). These ratios are widely used as descriptors of the chemical properties of HA [31]. The values of E_2_/E_3_ and E_4_/E_6_ are generally inversely correlated with the aromaticity, degree of condensation, and molecular weight of HA [32,33,34]. In our study, the E_2_/E_3_ and E_4_/E_6_ ratios decreased with the increase in pH (see Table 1). This finding suggests that sequential pH solubilization progressively leads to HA fractions having higher aromaticity content and, in line with the gel permeation experiments, a higher molecular weight distribution. It is interesting to note that HA exhibiting appreciable aromaticity and sizes are also likely associated with a high degree of hydrophobicity [35].

### 2.2. Sorption Kinetics of MB

#### 2.2.1. MB Sorption Kinetics on NYT

**Figure 2:**
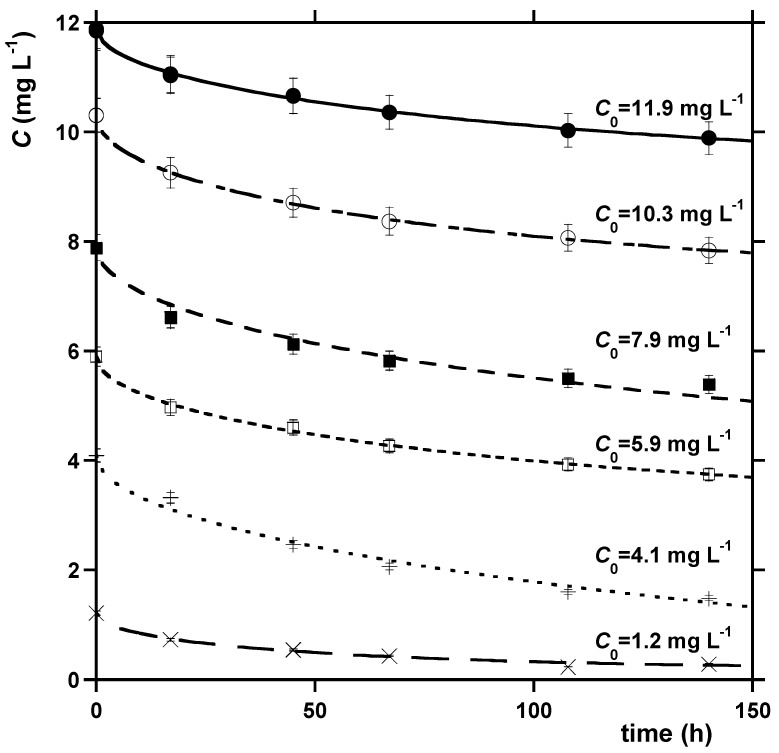
Dependence of the MB aqueous concentration on time in the presence of NYT; *C*_0_ = initial MB concentration in the liquid phase; *T* = 20 °C. The curves in the figure were obtained via non-linear regression of the data using the Vermeulen model.

Figure 2 shows the trend of the total MB concentration in the liquid phase versus time at different initial concentrations of MB and a constant loading of natural zeolite (NYT). For all the kinetic runs, an appreciable decrease in the MB concentration was observed as a result of the sorption process.

In order to model the uptake of MB, the popular pseudo-first-order (PFO), pseudo-second-order (PSO), Boyd, and Vermeulen models were selected.

The PFO model is an empirical model in which the rate of sorption is considered to be proportional to the distance from equilibrium, wherein the latter is expressed as the difference between the sorbed amount at equilibrium and at any time *t* [36]:

(1) $$\frac{dq}{dt}=k_{1}\left(q_{e}-q\right)$$

Here, $k_{1}$ (h^−1^) is the PFO kinetic rate constant, and q (mg g^−1^) is the amount of MB sorbed per mass of sorbent (see Equation (15)), wherein the subscript *e* denotes the sorption equilibrium state.

Another empirical model is the PSO model (Equation (2)), which considers the rate of sorption to be proportional to the square of the distance from equilibrium [36]:

(2) $$\frac{dq}{dt}=k_{2}{\left(q_{e}-q\right)}^{2}$$

Here, $k_{2}$ (g mg^−1^ h^−1^) is the PSO kinetic rate constant.

Integrating Equations (1) and (2) for the boundary conditions *t* = 0 to *t* and q = 0 to q and replacing q with Equation (15) permits us to derive the dependence of the MB aqueous concentration (mg L^−1^) on time, according to the PFO (Equation (3)) and PSO (Equation (4)) models, respectively:

(3) $$C=C_{0}-Xq_{e}\left(1-\exp\left(-k_{1}t\right)\right)$$

(4) $$C=C_{0}-X\frac{q_{e}{}^{2}k_{2}t}{1+q_{e}k_{2}t}$$

The Boyd (or Reichenberg) model [37] represents an approximated form of a more complex sorption kinetic model relying on the intraparticle diffusion theory developed, among others, by Boyd himself and Crank [38]. The Boyd approximating model consists of the following two equations.

(5) $$C=C_{0}-Xq_{e}\left(\frac{6}{\pi^{3/2}}\sqrt{Bt}-\frac{3}{\pi^{2}}Bt\right)\;\;\;\;(\mathrm{for}\;q/q_{e}<0.85)$$

(6) $$C=C_{0}-Xq_{e}\left(1-\frac{6}{\pi^{2}}\exp\left(-Bt\right)\right)\;\;\;\;(\mathrm{for}\;q/q_{e}>0.85)$$

Here, *B* (h^−1^) is a constant related to the sorbent particle size and the effective diffusion coefficient of the sorbate inside the sorbent.

Another approximation of the intraparticle diffusion model is the Vermeulen equation [39]:

(7) $$C=C_{0}-Xq_{e}\sqrt{\left(1-\exp\left(-k_{D}t\right)\right)}$$

The parameter $k_{D}$ (h^−1^) has a physical meaning similar to that of *B*.

Equations (3)–(7) were used to model the kinetic data for the sorption of MB onto NYT. The Boyd model was applied, switching from Equations (5) to (6) in correspondence with the threshold value *q*/*q_e_* = 0.85. Table 2 and Table S1 show the results of the regression procedure. According to a statistical analysis, the results of which are reported in these tables, in most of the cases, the sorption kinetics conform more closely to the Vermeulen and the Boyd models, as can be inferred from the low AICc values and the low error of the estimated parameters. Moreover, the Vermeulen equation provides a reliable estimate of the amount of MB sorbed at equilibrium.

The result of the application of the Vermeulen model to the kinetic data is presented in Figure 2. The close fitting between the Vermeulen model and the Boyd model could suggest that the sorption of MB onto NYT is controlled by intraparticle diffusion.

#### 2.2.2. MB Sorption Kinetics on NYT-HA

**Figure 3:**
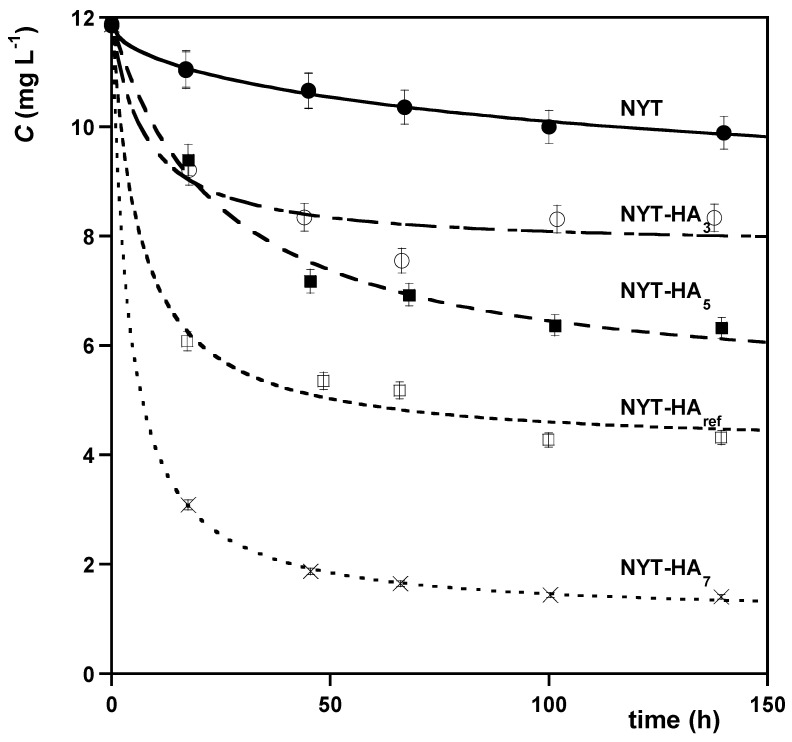
Comparison of the sorption kinetics regarding the sorption of MB onto various sorbents. Initial concentration of MB = 11.9 mg L^−1^; *T* = 20 °C. The curves in the figure were obtained via nonlinear regression analysis using the PSO model, with the exception of the NYT data set modelled using the Vermeulen equation.

The sorption rate of MB as affected by humic acids immobilization on the NYT surface is shown in Figure 3.

From the figure, it is evident that the sorption kinetics of MB are influenced by both the presence of HA and its pH fractionation. The uptake rate of MB decreases in the following row: NYT-HA_7_ > NYT-HA_ref_ > NYT-HA_5_ > NYT-HA_3_ > NYT.

The data in figure were modelled using Equations (3)–(7), and the results of this procedure are displayed in Table 3 and Table S2. Overall, the PSO model provides the best agreement with the experimental data, while the Boyd and Vermeulen intraparticle diffusion models were found to be less accurate than when they were used for modelling the sorption kinetics of MB onto NYT alone. This finding could be explained by the fact that the rate of MB uptake onto NYT-HA is controlled by surface interactions with the HA domain rather than by diffusion phenomena [40,41].

### 2.3. Sorption Equilibrium

#### 2.3.1. Isotherms of the Sorption of MB onto Natural Zeolite and Natural Zeolite–Humic Acids Adducts: Effect of pH Fractionation

**Figure 4:**
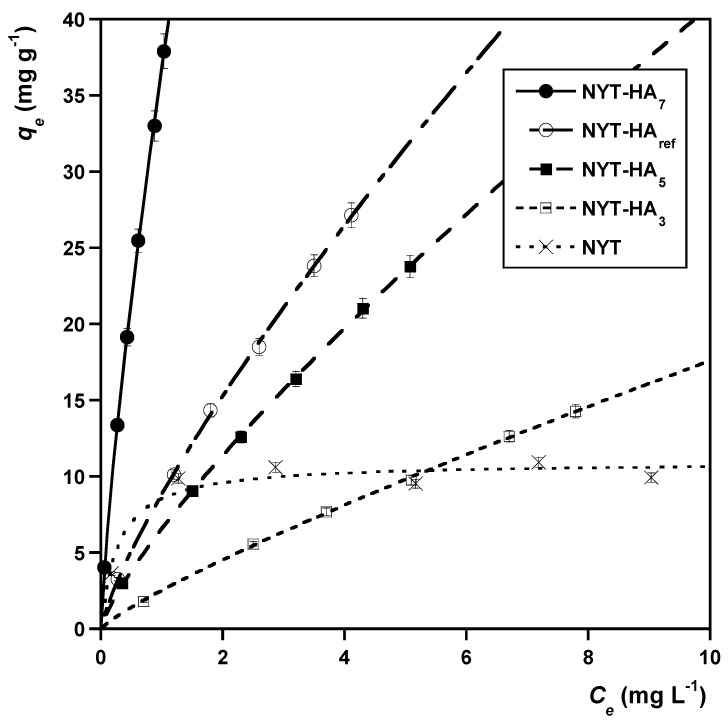
Sorption isotherms regarding the sorption of MB onto various sorbents. *T* = 20 °C. The curves in the figure were obtained via nonlinear regression analysis using the Freundlich model, with the exception of NYT data set modelled using the Langmuir equation.

Figure 4 compares the isotherm data at equilibrium for the sorption of MB onto various sorbents.

As can be seen, the raw zeolitic material has the lowest sorption capacity. It is clear that NYT benefits from the presence of HA and, more importantly, from its fractionation. NYT-HA_7_ outperforms all the other tested sorbents in terms of sorption efficiency, followed by NYT-HA_ref_, NYT-HA_5_, and NYT-HA_3_.

In order to shed light on the reasons underlying the different behaviors of the sorbents, their points of zero charge were measured and reported in Table 4.

The point of zero charge (pH_PZC_) is a useful parameter for gaining information on the net surface charge of a sorbent at a given pH and may help in elucidating mechanisms of sorption [42,43]. Generally, the higher or the lower the pH with respect to pH_PZC_, the greater the net negative or positive charge surface of the sorbent, respectively. According to the pH_PZC_ values reported in Table 4, NYT bears a net positive charge surface at the experimental pH (7.4). In contrast, all the NYT-HA samples have lower pH_PZC_ values and are negatively charged (pH_PZC_ < pH). The lower pH_PZC_ of the NYT-HA sorbents can be ascribed to the presence of acidic groups, mostly carboxylic and phenolic groups, in the HA moiety [44]. The observed pH_PZC_ decrement is less marked than that found for other materials coated with HA [44,45]. A feasible explanation for this finding is the partial decarboxylation of our HA during the thermal immobilization procedure [46,47].

In many studies, the sorption of MB is ascribed to electrostatic forces established between the negatively charged groups of the sorbent surface and the MB cation [48,49,50]. It is clear that such interactions are favored when pH_PZC_ < pH, and this would explain the lower uptake of MB by NYT as compared to the NYT-HA samples. However, the pH_PZC_ values of the NYT-HA materials are similar to each other and show a poor correlation with sorbent sorption capacity (cfs. Table 4 and Figure 4). This suggests that factors other than pH_PZC_ also control the performance of the sorbents. The results of the UV-Vis and GPC experiments support this hypothesis: in Section 2.1, it was shown that the HA fractions obtained at higher pH levels were larger and had higher aromaticity and hydrophobicity than those isolated at lower pH levels. Moreover, the sorbents containing higher amounts of pH-fractionated HA exhibited higher sorption capacity (Figure 4). Based on the above considerations, it is reasonable to infer that the sorption of MB onto the NYT-HA materials is primarily driven by π–π and hydrophobic effects. Similar interactions were found for MB in the presence of carbon nanotubes [51] and cyclodextrin derivatives [52]. The sorbing MB molecule is expected to orient itself parallel to the sorbent surface, with the positively charged sulfur atom facing the liquid phase [51].

The data in Figure 4 were modelled using the most representative sorption isotherm models, namely, the classical Langmuir model [53] and the Freundlich model [54,55]:

(8) $$q_{e}=\frac{q_{m}K_{L}C_{e}}{1+K_{L}C_{e}}$$

(9) $$q_{e}=K_{F}C_{e}{}^{n}$$

$q_{m}$ (mg g^−1^) and $K_{L}$ (L mg^−1^) represent the maximum sorption capacity and the equilibrium constant of the Langmuir model, whereas $K_{F}$ (mg^1-*n*^ g^−1^ L*^n^*) and n (dimensionless) are the Freundlich isotherm parameters. The results of the fitting procedure are displayed in Table 5. They show that the NYT isotherm data more closely fit the Langmuir model, while the HYT-HA isotherms are better described by the Freundlich model. These findings may be taken as an indication that NYT-HA, contrary to NYT, exhibits a heterogeneous sorbing surface for MB [56].

In order to evaluate the efficiency of the best sorbent, NYT-HA_7_, Table 6 reports the predicted amounts of MB sorbed ($q_{e}$) at the highest equilibrium concentration explored (*C_e_* = 1.0 mg L^−1^) and, for comparison, the predicted amounts of MB sorbed in the same experimental conditions by other sorbents from the literature

The data in Table 6 reveal that the performance of NYT-HA_7_ is quite good in comparison with that of other materials. The sorption properties of the NYT-HA material could be improved by enhancing the loading content of humic acids and varying their chemical compositions, for example, by using a different source of humic acids or via physicochemical activation. These aspects will be explored in a more systematic manner in future works.

#### 2.3.2. Sorption Thermodynamics

**Figure 5:**
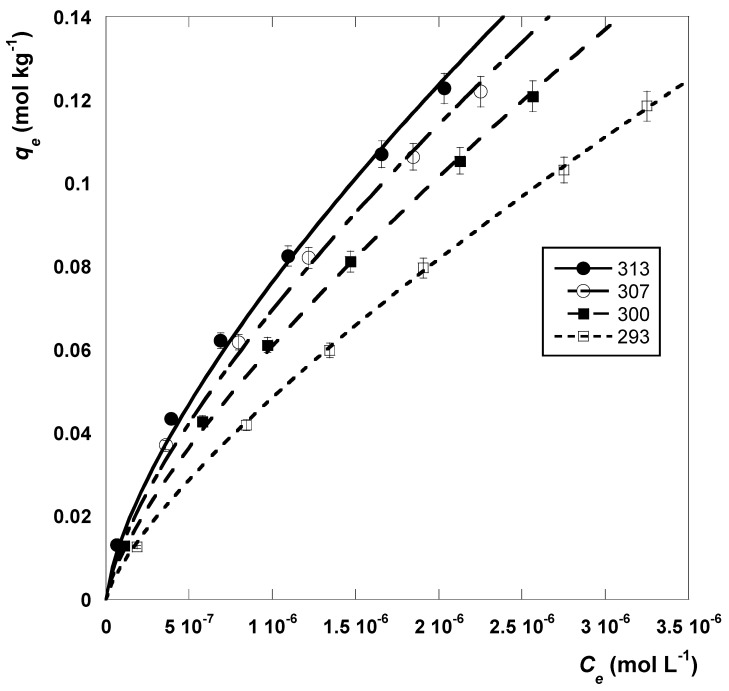
Sorption isotherms regarding the sorption of MB onto NYT-HA_7_ at various temperatures. The curves in the figure were obtained via non-linear regression analysis using the Freundlich isotherm model.

The effect of temperature on the sorption equilibrium of MB was investigated for the best-performing sorbent NYT-HA_7_. The sorption isotherms at 293, 300, 307, and 313 K are displayed in Figure 5. The curves in the figure were obtained by applying the Freundlich model, and the estimates of the fitted parameters are reported in Table 7. The values of $q_{e}$ and $C_{e}$ in Figure 5 were expressed in mol kg^−1^ and mol L^−1^ in accordance with the standard states universally accepted in thermodynamic sorption studies [68].

It can be seen from the figure that the sorption of MB increased with temperature, which suggests that the process is endothermic in nature (ΔH°>0). A similar behavior was found for the sorption of MB onto bentonite [69], peanut-shell-based activated carbon [70], and iron-oxide-modified montmorillonite [71]. In contrast, an exothermic sorption of MB was observed, for example, in the presence of bone char [72], citrate-modified pomelo peel [73], and a magnetic-gas-to-liquid-derived biosolid [74].

In order to acquire quantitative information on the thermodynamic parameters of the sorption of MB onto NYT-HA_7_, the following expressions were used [75]:

(10) ΔG°=−RTlnK°

(11) ΔG°=ΔH°−TΔS°

Here, R = 8.314 J K^−1^ mol^−1^ is the universal gas constant, and ΔG° (kJ mol^−1^), ΔH° (kJ mol^−1^), and ΔS° (J K^−1^ mol^−1^) are the standard sorption Gibbs energy, standard sorption enthalpy, and standard sorption entropy, respectively. K° (dimensionless) is the thermodynamic sorption equilibrium constant [76]. Its value should be derived from the best-fitting isotherm model. In the case of the Freundlich model, K° can be calculated from its exponent n, as recently shown by Debord et al. [56]. According to the authors, the Freundlich isotherm is consistent with the existence of a heterogeneous sorbent whose sorbing sites exhibit an exponential distribution of binding energies (ΔG°). The sorption equilibrium on each site can be locally described using a Langmuir-type model. The exponent n is related to the mean value of ΔG° ($\Delta G{{}^{\circ}}_{0}$) and to its associated thermodynamic equilibrium constant $K{{}^{\circ}}_{0}$ via the relationships

(12) $$\Delta G{{}^{\circ}}_{0}=-\frac{RT}{n}$$

(13) $$K{{}^{\circ}}_{0}=\exp\left(\frac{1}{n}\right)$$

from which we can obtain

(14) $$\frac{1}{n}=-\frac{\Delta H{}^{\circ}}{RT}+\frac{\Delta S{}^{\circ}}{R}$$

According to Equation (14), the values of ΔH° and ΔS° (which are assumed not to vary appreciably over the temperature range investigated) can be estimated from the slope and the intercept, respectively, with the y-axis of the straight line obtained by plotting 1/n against 1/T.

**Figure 6:**
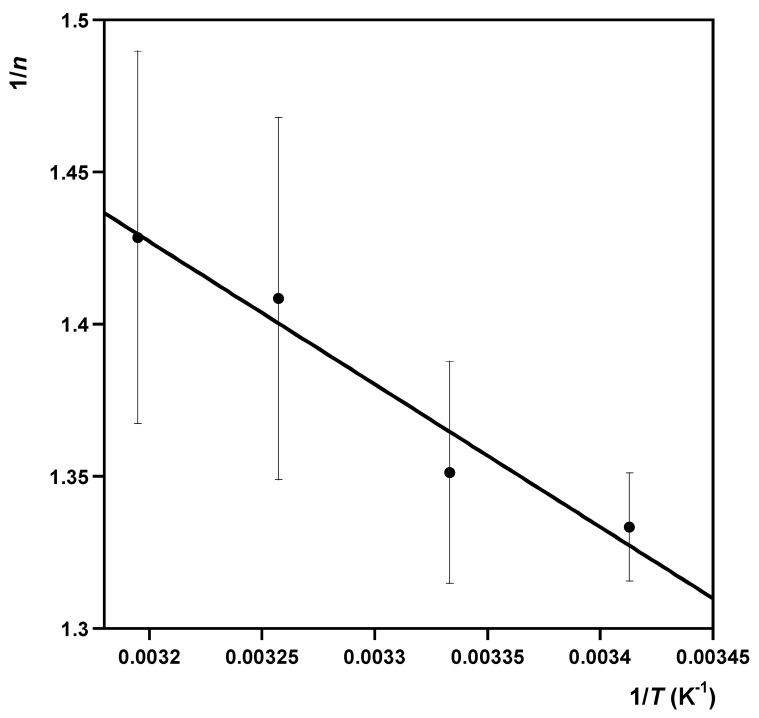
Plot of 1/*n* against 1/*T* for the estimation of the standard sorption enthalpy (ΔH°) and the standard sorption entropy (ΔS°).

The results of the application of Equation (14) to the sorption data regarding the sorption of MB onto NYT-HA_7_ are graphically displayed in Figure 6. The fitting results exhibit a good linear relationship, and the resultant values of ΔH° and ΔS° were 3.9 ± 0.6 kJ mol^−1^ and 24 ± 2 J K^−1^ mol^−1^, respectively.

## 3. Materials and Methods

### 3.1. Chemicals

All reagents were purchased from Sigma-Aldrich (St. Louis, MO, USA).

### 3.2. Natural Zeolite Sample

The natural zeolite sample came from a Neapolitan yellow tuff quarry located in Marano, some 10 km NW of Naples, Italy. The material was characterized in a previous work [77] as being composed 34% by weight of zeolite (phillipsite). Its chemical composition is as follows, wt%: SiO_2_ = 52.9, Al_2_O_3_ = 14.7, Fe_2_O_3_ = 4.0, MgO = 1.1, CaO = 2.1, K_2_O = 7.6, Na_2_O = 2.8, and P_2_O_5_ = 0.1. The corresponding cation exchange capacity and specific surface area are 1.90 meq g^−1^ and 23 m^2^ g^−1^, respectively.

Before its use, the material (henceforth termed NYT, denoting “Neapolitan yellow tuff”) was crushed and sieved to produce particles with a size (0.5–1 mm) suitable for real industrial applications [78]. Afterwards, the cation exchange surface of the sample was saturated in Ca^2+^ via treatment with 3 M of CaCl_2_ for 8 h under gentle stirring at a mass/liquid ratio of 1:100. The procedure was repeated two more times by replacing the supernatant with a fresh CaCl_2_ solution; finally, the NYT sample was recovered, washed with pure water, and dried at 40 °C in an oven for 24 h.

### 3.3. Extraction of Humic Acids from Vegetable Compost, pH Fractionation, and Characterization

#### 3.3.1. HA Extraction

A commercial vegetable compost (VC) purchased from Selex (Milan, Italy) was used as source of humic acids (HA). The total organic carbon and the humic + fulvic carbon content of VC were (as declared by the supplier) 22% and 3% (in dry weight), respectively. HA were extracted from VC using NaOH and sodium pyrophosphate (Na_4_P_2_O_7_) [77]. In more detail, 1 kg of VC was placed in contact, for 2 days under stirring, with 10 L of 0.1 M NaOH + 0.1 M Na_4_P_2_O_7_. The sample was centrifuged at 1000 *g*-force units of relative centrifugal force for 1 h; then, the supernatant was recovered, and its pH was adjusted to 1.5 with a few drops of concentrated HCl in order to promote HA precipitation. The suspension was stored at 4 °C for 2 days and then centrifuged at 1000 *g*-force units for 1 h. Finally, the precipitate was collected, purified in a dialysis bag (cut off = 1000 Da), and lyophilized.

#### 3.3.2. pH-Fractionation of HA

The HA extracted from VC (unfractionated HA soluble at pH 7, HA_ref_) was separated into three fractions differing in their pH solubility. A total of 1 g of HA_ref_ was placed in contact with 1 L of pure water, and the pH was adjusted to 3.0 using NaOH. After 24 h, the soluble part of HA at pH 3 (hereafter referred to as HA_3_) was recovered from the supernatant via acidic precipitation. The insoluble part was adjusted to pH 5 and then pH 7. The soluble fractions of HA at pH 5 and 7 were recovered through the same procedure as that used for HA_3_ and denoted as HA_5_ and HA_7_, respectively.

#### 3.3.3. HA Characterization

HA were analyzed via gel permeation chromatography (GPC) using a Waters instrument consisting of a 515 HPLC pump and a 2487 dual λ absorbance detector; additionally, the instrument was equipped with a Biosep (Melton Mowbray, UK) Sec-2000 (300 × 7.80 mm^2^) column. According to a previous report [79], the following operational conditions were selected: mobile phase composition = 75% 10 mM phosphate buffer (pH 7.0) + 25% CH_3_CN (*v*/*v*); flow rate = 0.7 mL min^−1^; detection wavelength = 280 nm.

UV-Vis spectroscopic characterization of HA solutions was carried out by determining the ratio between the absorbance at 250 nm and 365 nm (E_2_/E_3_) and at 465 nm and 665 nm (E_4_/E_6_) [80] via a Perkin-Elmer (Waltham, MA, USA) Lambda 40 spectrophotometer.

### 3.4. Preparation of the Humic Acids—Zeolitic Tuff Sorbents and Point-of-Zero-Charge Measurements

Each HA sample (unfractionated or pH-fractionated) was dissolved in 10 mM Tris buffer (pH ≈ 7.4) to obtain a 100 mg L^−1^ solution. In total, 40 mL of each solution was then placed with 1 g of NYT pre-treated with CaCl_2_ in 50 mL conical tubes under continuous stirring using an orbital shaker operating at 30 rpm and under room temperature. At selected times, the absorbance of the supernatant at 450 nm was measured for the quantification of the loaded HA. When the loading of HA onto NYT reached approximatively the value of 2 mg g^−1^, the suspension was filtered on filter paper with a vacuum pump, and the solid was recovered and heated at 330 °C in an oven to stabilize, via decarboxylation, the NYT-HA adduct [17]. The entire procedure, i.e., HA loading and immobilization, was repeated four more times until reaching, for each type of sorbent, an HA loading of about 10 mg g^−1^. The obtained materials were named NYT-HA_ref_, NYT-HA_3_, NYT-HA_5_, and NYT-HA_7_.

The point of zero charge (pH_PZC_) was determined via a pH titration procedure [42,43]. Aliquots of 0.01 M NaCl solution were placed in contact with 0.29 g L^−1^ of sorbent, and the pH was adjusted to between 2 and 10. The pH was measured again after 2 days. A plot of the final pH against the initial pH was constructed. The pH at which the initial and final pH values were the same corresponded to pH_PZC._

### 3.5. MB Spectrophotometric Measurements and Calibration Curve

During preliminary experiments, we observed that MB is prone to strongly sorbing onto any glass surface, including vials and Pasteur pipettes but also quartz cuvettes, in line with a previous report [81]. This can lead to an overestimation of the sorption capacity of NYT and HA for MB. For this reason, we used only plastic materials to store, handle, and analyze the MB solutions.

The MB solutions were analyzed in polystyrene cuvettes using a Lambda 40 Perkin Elmer spectrophotometer. The absorbance recorded at 665 nm was used for determining the MB concentration.

The absorbance–concentration calibration curve was constructed as follows. A 100 mg L^−1^ stock solution of MB (methylene blue, also known as methylthioninium chloride, with a molar mass = 319.85 g mol^−1^) was prepared by dissolving 58.44 mg of methylene blue trihydrate (molar mass = 373.9 g mol^−1^) into 0.5 L of an aqueous solution buffered at pH = 7.4 with 10 mM Tris buffer. Appropriate volumes of this working solution were diluted with the addition of water in order to obtain a set of MB solutions with concentrations ranging from 0.5 mg L^−1^ to 14 mg L^−1^. The absorbance at 665 nm of each solution was then measured and plotted as a function of the MB concentration. The calibration curve (see Figure S1 in the Supplementary Information) was obtained by fitting the absorbance vs. concentration data with Equation (S6). The latter equation considers that MB exists in monomeric and dimeric forms only (a valid assumption for the range of concentrations used). More specific details about the calculation method can be found in the Supplementary Information.

### 3.6. MB Sorption Experiments

A total of 12 mg of the selected sorbent was placed in 50 mL polypropylene conical tubes. Afterwards, 42 mL of an MB aqueous solution (buffered at pH = 7.4) with a concentration varying between 1 and 12 mg L^−1^ (a concentration range similar to that reported in other MB sorption studies [60,82,83]) was added. The samples were thermostated at the desired temperature (20, 27, 34, or 40 °C) under continuous stirring and periodically analyzed via spectrophotometric measurements at 665 nm. The amount of MB sorbed per mass of sorbent (q, mg g^−1^) was calculated using the following mass balance formula:

(15) $$q=\frac{C_{0}-C}{X}$$

Here, $C_{0}$ and C (mg L^−1^) are the initial and actual concentrations in the liquid phase, respectively, whereas X (g L^−1^) represents the ratio between the mass of sorbent and the volume of the solution.

## 4. Conclusions

In this work, HA were immobilized onto natural zeolites and successfully applied in the removal of MB from the liquid phase. The sorbing ability of HA is well known in the literature. Here, we showed that the fractionation of HA can greatly enhance their sorption efficiency. This can be easily accomplished via a sequential pH solubilization procedure. Thanks to their chemical heterogeneity, HA can interact with other molecules through different mechanisms involving electrostatic and π–π interactions and hydrophobic effects. Each HA fraction exhibits distinctive chemical characteristics that make it more or less affine towards a specific group of pollutants. Depending on the chemical nature of the target pollutant, the more appropriate fraction of HA can be conveniently selected to prepare an efficient sorbent material.

Based on the above considerations, natural zeolites modified with pH-fractionated humic acids can be considered versatile and promising materials for water remediation processes. Future works will be devoted to exploring the possibility of further implementing the sorption capacity of the HA-based sorbents, for example, by increasing the HA content and using HA of different origins and altering their chemical surface activity.

## Figures and Tables

**Table 1:** Optical properties of HA samples as determined via the E_2_/E_3_ and E_4_/E_6_ indices.

HA Type	E_2_/E_3_	E_4_/E_6_
HA_ref_	1.7	4.0
HA_3_	1.9	4.4
HA_5_	1.8	4.1
HA_7_	1.6	3.6

**Table 2:** Kinetic parameters for the sorption of MB onto NYT. Initial aqueous concentration of MB = 11.9 mg L^−1^; sorbent dosage = 0.29 g L^−1^; pH = 7.4; *T* = 20 °C (additional results can be found in Table S1).

Model	*k*_1_ (h^−1^)	*k*_2_ (g mg^−1^ h^−1^)	*B* (h^−1^)	*k_D_* (h^−1^)	Res. Sum of Squares	AICc
PFO	0.023 ± 0.003	-	-	-	1.087	−8.23
PSO	-	0.0027 ± 0.0005	-	-	0.533	−14.63
Boyd	-	-	0.0036 ± 0.0009	-	0.093	−30.36
Vermeulen	-	-	-	0.0048 ± 0.0008	0.094	−30.25

**Table 3:** Kinetic parameters for the sorption of MB onto NYT-HA_7_: initial aqueous concentration of MB = 11.9 mg L^−1^; sorbent dosage = 0.29 g L^−1^; pH = 7.4; *T* = 20 °C (additional results are reported in Table S2).

Model	*k*_1_ (h^−1^)	*k*_2_ (g mg^−1^ h^−1^)	*B* (h^−1^)	*k_D_* (h^−1^)	Res. Sum of Squares	AICc
PFO	0.108 ± 0.008	-	-	-	1.241	23.03
PSO	-	0.0066 ± 0.0003	-	-	0.085	9.65
Boyd	-	-	0.077 ± 0.006	-	0.728	20.36
Vermeulen	-	-	-	0.070 ± 0.005	0.616	19.53

**Table 4:** Points of zero charge (pH_PZC_) of the sorbent materials.

Sorbent	pH_PZC_
NYT	7.2
NYT-HA_ref_	5.0
NYT-HA_3_	4.1
NYT-HA_5_	4.9
NYT-HA_7_	4.8

**Table 5:** Isotherm sorption parameters for the uptake of MB onto NYT and NYT-HA at 20 °C.

Sorbent	$q_{m}$ (mg g^−1^)	$K_{L}$ (L mg^−1^)	Res. Sum of Squares	AICc	$K_{F}$ (mg^1-*n*^ g^−1^ L*^n^*)	n	Res. Sum of Squares	AICc
NYT	10.9 ± 0.5	4 ± 1	2.930	13.70	8 ± 1	0.18 ± 0.08	11.781	22.04
NYT-HA_ref_	70 ± 10	0.14 ± 0.03	1.794	10.76	8.8 ± 0.1	0.79 ± 0.01	0.190	−2.73
NYT-HA_3_	44 ± 8	0.06 ± 0.02	0.477	2.81	2.52 ± 0.07	0.84 ± 0.02	0.078	−8.08
NYT-HA_5_	70 ± 10	0.10 ± 0.02	1.159	8.14	6.56 ± 0.08	0.79 ± 0.01	0.076	−8.19
NYT-HA_7_	110 ± 10	0.5 ± 0.1	1.319	8.91	36.7 ± 0.2	0.77 ± 0.01	0.255	−0.95

**Table 6:** Predicted sorption capacity (*q_e_*) of various sorbents for MB in correspondence with an equilibrium aqueous concentration of MB equal to 1.0 mg L^−1^.

Sorbent	Predicted $q_{e}$ (mg g^−1^)	pH ^a^	Sorbent Dosage (g L^−1^*)*	*T* (K)	Fitting Model	Ref.
NYT-HA_7_	37	7.4 (buff.)	0.3	293	Freundlich	This work
Jujube-stone-based activated carbon	23	7.0	1	298	Langmuir	[57]
Kaolin	19	2.0	0.5	298	Langmuir	[58]
Zeolite waste	8	free	1	298	Langmuir	[59]
Carbon nanotubes	8	7.0	0.3	298	Langmuir	[60]
Modified lignocellulosic materials	39	6.0	10	298	Langmuir	[61]
Row date pits	11	8.0	5	298	Langmuir	[62]
Graphene oxide	191	7.0	0.5	298	Langmuir	[63]
Silica gel/eggshell powder	16	7.0	0.25	298	Freundlich	[64]
Magnetic activated biochar nanocomposites derived from wakame	183	n.d.	1	293	Langmuir	[49]
Commercial activated carbon	24	6.9	4	297	Langmuir	[65]
Surfactant-modified activated carbon	106	5.0	0.15	298	Langmuir	[66]
ZnCl_2_-Activated Carbon	642	free	0.5	303	Langmuir	[67]

^a^ (buff.) = buffered solution; free = natural pH of the mixture, not adjusted; n.d. = no data available.

**Table 7:** Freundlich isotherm and thermodynamic parameters for the sorption of MB onto NYT-HA_7_ at various temperatures.

T (K)	$K_{F}$ (mol^1−*n*^ kg^−1^ L^*n*^)	n	$\Delta G{{}^{\circ}}_{0}$ (kJ mol^−1^)	ΔH° (kJ mol^−1^)	ΔS° (J K^−1^ mol^−1^)
293	1600 ± 300	0.75 ± 0.01	−3.25 ± 0.04	3.9 ± 0.6	24 ± 2
300	1600 ± 400	0.74 ± 0.02	−3.37 ± 0.09		
307	1400 ± 600	0.71 ± 0.03	−3.6 ± 0.2		
313	1200 ± 500	0.70 ± 0.03	−3.7 ± 0.2		

## Data Availability

Data are available from the author on request.

## Supplementary Materials

Please add this part as following format: ‘The following supporting information can be downloaded at: https://www.mdpi.com/article/10.3390/molecules28207083/s1. Refs [84,85] are in the Supplementary Materials.
