# Supplementary material for: Enhanced Sorption Performance of Natural Zeolites Modified with pH-Fractionated Humic Acids for the Removal of Methylene Blue from Water
Source: Molecules. 2023 Oct 14;28(20):7083. doi: 10.3390/molecules28207083 (PMC10609103; doi:10.3390/molecules28207083)
Supplement: Supplementary file 1 [file molecules-28-07083-s001.zip › molecules-2594837-supplementary.pdf]

Supporting Information

Enhanced sorption performances of natural zeolites modified with pH-fractionated humic acids for methylene blue water removal

Stefano Salvestrini [1,*], Jean Debord [2] and Jean-Claude Bollinger [3]

[1] Department of Environmental, Biological and Pharmaceutical Sciences and Technologies, University of Campania “Luigi Vanvitelli”, 81100 Caserta, Italy; stefano.salvestrini@unicampania.it
[2] Service de Pharmacologie-Toxicologie, Hôpital Dupuytren, 87042 Limoges, France; jean.debord@unilim.fr
[3] Université de Limoges, Laboratoire E2Lim, Faculté des Sciences et Techniques, 87060 Limoges, France; jean-claude.bollinger@unilim.fr

* Correspondence: stefano.salvestrini@unicampania.it; Tel.:++39(0)823274639

S1. Calibration curve and determination of the dimerization equilibrium constant

Owing to its cationic nature, MB tends to self-associate forming a variety of aggregates including dimers, trimers and tetramers. At MB concentration lower than 70 ppm, the presence of trimeric and tetrameric forms can be neglected [1] and the reaction scheme reduces to:

$$2MB \leftrightharpoons (MB)_2 \qquad \text{(S1)}$$

where $(MB)_2$ is the dimeric form of MB.

Let C_m and C_d represent the concentration (mol L^{-1} also noted M) of MB in monomeric and dimeric form, respectively. The dimerization equilibrium constant (K) is given by:

$$K = \frac{C_d}{C_m^2} \qquad \text{(S2)}$$

Moreover, the total concentration of MB (C) can be expressed as:

$$C = C_m + 2C_d = C_m + 2KC_m^2 \qquad \text{(S3)}$$

The quadratic equation in C_m admits the following positive root:

$$C_m = \frac{-1+\sqrt{1+8KC}}{4K} \tag{S4}$$

According to the Lambert-Beer law, the total absorbance (A) of the solution by MB species can be written as:

$$A = \epsilon_m l C_m + \epsilon_d l C_d = \epsilon_m l C_m + \epsilon_d l \frac{C-C_m}{2} \tag{S5}$$

where ϵ_m and ϵ_d are the molar extinction coefficients (M^{-1} cm^{-1}) of the monomer and dimer, respectively, whereas l (cm) is the optical length.

Using eq. (S4), we get the absorbance A as a function of the total concentration C:

$$A = \left(\epsilon_m - \frac{\epsilon_d}{2}\right) l \cdot \frac{-1+\sqrt{1+8KC}}{4K} + \frac{\epsilon_d l C}{2} \tag{S6}$$

Note that, due to a typing error, a slightly different equation was published by Spencer and Sutter [2]. This equation cannot be inverted analytically to determine the total MB concentration C from absorbance measurements, but it can be solved with respect to C_m using the definition of K:

$$A = \epsilon_m l C_m + \epsilon_d l K C_m^2 \tag{S7}$$

The quadratic equation in C_m admits the following positive root:

$$C_m = \frac{-\epsilon_m l + \sqrt{(\epsilon_m l)^2 + 4\epsilon_d l K A}}{2\epsilon_d l K} \tag{S8}$$

Equation (S8) allows to calculate C_d and C by means of Equations (S2) and (S5), respectively.

The calibration curve was obtained fitting the A vs C experimental data with Equation (S6) by non-linear regression analysis and the results are graphically displayed in Figure S1. The estimates of ε_m, ε_d *and* K at T = 20 °C were found to be (8.04 ± 0.01) × 10^{-4} M^{-1} cm^{-1}, (4.0 ± 0.1) 10^{-4} M^{-1} cm^{-1} and (7.38 ± 0.01) × 10^{-3} M^{-1},

respectively; these values are in very good agreement with those reported in recent literature at similar temperature and wavelength [1]. For absorbance measurements at $T \neq 20$ °C, the calibration curve was corrected taken the values of K from the literature [1].

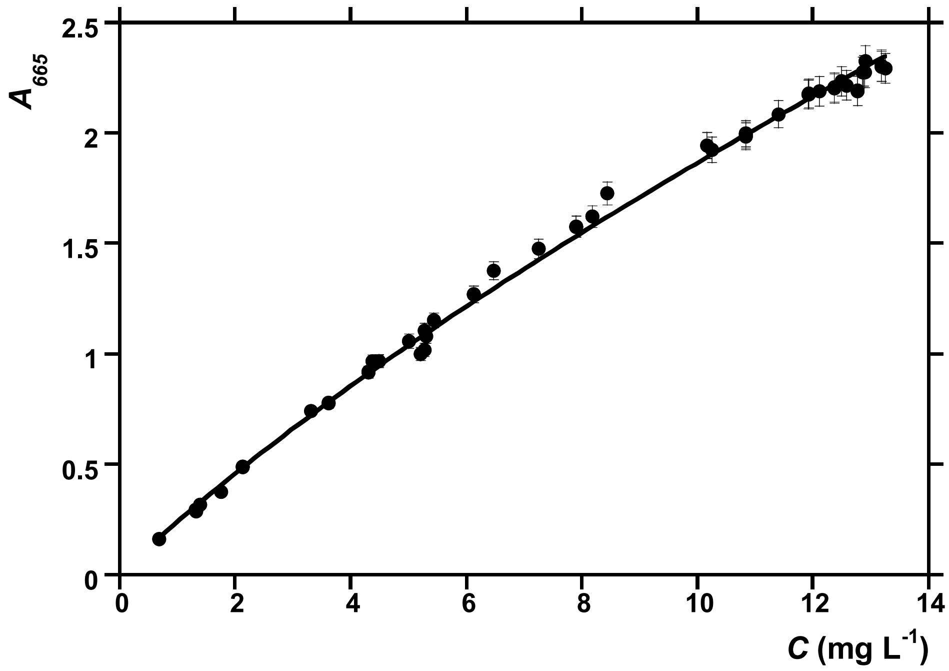

Figure S1. Plot of the absorbance at 665 nm (A_{665}) vs the total MB concentration in solution (C) at T = 20 °C. The curve in figure was obtained via nonlinear curve-fitting using Equation (S6).

S2. Sorption modelling

Table S1. Kinetic parameters for the MB sorption onto NYT.

**Model**	k_1 (h^{-1})	k_2 (g mg^{-1} h^{-1})	B (h^{-1})	k_D (h^{-1})	**Res. sum of squares**	**AICc**
			C_0=11.9 mg L^{-1}			
PFO	0.023 ± 0.003	-	-	-	1.087	-8.23
PSO	-	0.0027	-	-	0.533	-14.63

		± 0.0005				
Boyd	-	-	0.0036 ± 0.0009	-	0.093	-30.36
Vermeulen	-	-	-	0.0048 ± 0.0008	0.094	-30.25
C_0=10.3 mg L^{-1}						
PFO	0.025 ± 0.003	-	-	-	1.314	-6.51
PSO	-	0.0025 ± 0.0003	-	-	0.438	-16.41
Boyd	-	-	0.0061 ± 0.0007	-	0.056	-34.96
Vermeulen	-	-	-	0.0070 ± 0.0005	0.044	-37.11
C_0=7.9 mg L^{-1}						
PFO	0.035 ± 0.004	-	-	-	1.977	-2.84
PSO	-	0.0042 ± 0.0006	-	-	0.568	-14.06
Boyd	-	-	0.0135 ± 0.0009	-	0.092	-30.42
Vermeulen	-	-	-	0.013 ± 0.001	0.151	-26.01
C_0=5.9 mg L^{-1}						
PFO	0.023 ± 0.003	-	-	-	1.699	-4.21
PSO	-	0.0025 ± 0.0006	-	-	0.901	-9.92

Boyd	-	-	0.004 ± 0.001	-	0.164	-25.26
Vermeulen	-	-	-	0.005 ± 0.001	0.192	-23.81
C_0=4.1 mg L^{-1}						
PFO	0.0192 ± 0.0001	-	-	-	0.020	-44.35
PSO	-	0.0013 ± 0.0001	-	-	0.195	-23.69
Boyd	-	-	0.0002 ± 0.0011	-	1.810	-3.64
Vermeulen	-	-	-	0.002 ± 0.002	1.720	-4.09
C_0=1.2 mg L^{-1}						
PFO	0.037 ± 0.005	-	-	-	0.362	-18.11
PSO	-	0.012 ± 0.001	-	-	0.172	-24.84
Boyd	-	-	0.014 ± 0.003	-	0.104	-29.37
Vermeulen	-	-	-	0.013 ± 0.002	0.111	-28.79

Table S2. Kinetic parameters for the MB sorption onto different sorbents; initial aqueous concentration of MB = 11.9 mg L^{-1}; sorbent dosage = 0.29 g L^{-1}; pH = 7.4.

**Model**	k_1 **(h^{-1})**	k_2 **(g mg^{-1} h^{-1})**	B **(h^{-1})**	k_D **(h^{-1})**	**Res. sum of squares**	**AICc**

NYT						
PFO	0.023 ± 0.003	-	-	-	0.090	-37.11
PSO	-	0.0027 ± 0.0005	-	-	0.044	-44.37
Boyd	-	-	0.0036 ± 0.0009	-	0.093	-30.36
Vermeulen	-	-	-	0.0048 ± 0.0008	0.094	-30.25
NYT-HA_{ref}						
PFO	0.09 ± 0.02	-	-	-	10.543	33.73
PSO	-	0.005 ± 0.002	-	-	4.555	29.53
Boyd	-	-	0.05 ± 0.02	-	7.988	32.34

Vermeulen	-	-	-	0.05 ± 0.01	7.696	32.16
			NYT-HA$_3$			
PFO	0.07 ± 0.02	-	-	-	5.415	30.40
PSO	-	0.009± 0.008	-	-	7.796	32.22
Boyd	-	-	0.05 ± 0.03	-	6.240	31.11
Vermeulen	-	-	-	0.05 ± 0.02	6.348	31.19
			NYT-HA$_5$			
PFO	0.035 ± 0.003	-	-	-	1.016	22.03
PSO	-	0.0017 ± 0.0005	-	-	2.877	27.24
Boyd	-	-	0.017	-	5.565	30.54

			± 0.007			
Vermeulen	-	-	-	0.015 ± 0.006	4.872	29.87
			NYT-HA$_7$			
PFO	0.108 ± 0.008	-	-	-	1.241	23.03
PSO	-	0.0066 ± 0.0003	-	-	0.085	9.65
Boyd	-	-	0.077 ± 0.006	-	0.728	20.36
Vermeulen	-	-	-	0.070 ± 0.005	0.616	19.53

References

1. Fernández-Pérez, A.; Valdés-Solís, T.; Marbán, G. Visible light spectroscopic analysis of Methylene Blue in water; the resonance virtual equilibrium hypothesis. Dyes Pigments 2019, 161, 448-456, doi:10.1016/J.DYEPIG.2018.09.083.

2. Spencer, W.; Sutter, J.R. Kinetic study of the monomer-dimer equilibrium of methylene blue in aqueous solution. J Phys Chem 1979, 83, 1573–1576, doi: 10.1021/j100475a004
